# Dose-dependent acute toxicity of chitosan nanoparticles with dual assessment of multisystem toxicopathology and oxidative stress biomarkers in Nile tilapia

**DOI:** 10.1038/s41598-026-54988-x

**Published:** 2026-06-04

**Authors:** Abdelaziz Rabea, Iman Ibrahim, Hebatallah Mahgoub, Ahmed El-Shaieb, Abdelaziz Mohamed, Mohamed Aboelmaati Rabea

**Affiliations:** Pathology Department, Faculty of Veterinary Medicine, Mansoura University, Mansoura, 35516 Egypt

**Keywords:** Nile tilapia (*Oreochromis niloticus*), Nano-chitosan, Neurotoxicity, Histopathology, Oxidative stress, Nanotoxicity, Biochemistry, Environmental sciences, Zoology

## Abstract

Nano-chitosan (nCS) is widely used in aquaculture for its antimicrobial and immunostimulatory properties, but its toxicological profile in fish remains poorly understood. This study assessed the dose-dependent acute toxic effects of nCS in Nile tilapia on oxidative stress biomarkers and multisystemic histopathology to clarify the mechanisms of systemic injury. Healthy fish (*n* = 80) were exposed to spherical nCS (8–20 nm (at concentrations of 0, 5, 10, and 20 mg/L for 96 h. Exposure to nCS caused dose-dependent toxicity. While no mortality was observed at concentrations up to 5 mg/L, the 20 mg/L dose induced significant behavioral changes and 50% mortality within 96 h. Biochemical analysis indicated systemic oxidative stress, with increased lipid peroxidation and depleted antioxidant defenses across the gills, kidney, liver, and muscle. Comprehensive histopathological examination of eight distinct tissues confirmed severe tissue damage, including branchial lamellar fusion, renal tubular necrosis, and neurotoxic effects in the brain, correlating with neurobehavioral distress. These findings indicate that acute exposure to nCS concentrations of ≥ 10 mg/L induces severe multisystemic necrosis, establishing a no-observed-adverse-effect-level (NOAEL) below 5 mg/L. The study identifies the gills and kidney as key nanotoxicity biomarkers, suggesting that strict application limits are essential to balance the therapeutic benefits and ecological risks of nCS in aquaculture.

**Supplementary Information:**

The online version contains supplementary material available at 10.1038/s41598-026-54988-x.

## Introduction

Aquaculture plays a vital role in the global food industry by providing a valuable protein source for human consumption^1^. Nile tilapia is the most important species in this industry due to its high productivity, rapid growth, adaptability, and resistance to disease and various environmental conditions^2^. To face a growing need for sustainable and effective production, the industry is turning to innovative technologies such as nanomaterial^3^.

Nanotechnology offers unique features that are useful in applications such as drug delivery and water purification due to their large surface area and high reactivity^4^, providing significant benefits for improved nutrient absorption and disease resistance in aquaculture^5^. Among the various nanomaterials, nano-chitosan (nCS) has demonstrated particular effectiveness, offering improved bioavailability, cellular uptake, and the ability to boost immunity and antioxidant defenses in fish, addressing the need for safe and cost-effective solutions^6^. nCS is derived from chitosan (CS), a natural polysaccharide that is biodegradable and biocompatible, obtained from chitin found in the shells of crustaceans like shrimp^7^. They are widely used in various aquaculture applications due to their antimicrobial, immunostimulatory and growth-promoting effects^8,9^^10^.

Although most studies have demonstrated the beneficial effects of nCS in fish^11^, there remains a significant knowledge gap regarding their dose-dependent toxicity, especially at higher concentrations and when introduced directly into the aquatic environment through water immersion^12^. Furthermore, understanding toxicity requires analyzing cellular and tissue-level responses, not just mortality^13^.

Acute toxicity tests determine the 96-hour lethal concentration^14^. In the present study, nCS concentrations of 0, 5, 10, and 20 mg/L were evaluated. However, mortality data alone are insufficient for a comprehensive toxicity profile. Evaluating sub lethal effects at the cellular and tissue levels is critical to ensure safety standards and understand the physiological impact of these nanoparticles^15^.

Therefore, we hypothesized that direct waterborne exposure to ultra-small nCS could induce concentration-dependent oxidative stress and multisystem toxicity in Nile tilapia. This study aimed to conduct a comprehensive assessment by quantifying oxidative stress markers, characterizing dose-dependent histopathological alterations across multiple organ systems, including investigating potential neurotoxicity and brain involvement, and establishing a practical safety threshold specifically for waterborne aquaculture application. This research seeks to provide novel insights by shifting the focus from the well-documented safe dietary use of nCS to exploring its potential waterborne toxicity, thereby balancing its therapeutic benefits with its ecological risks through a focus on both toxicological mechanisms and practical safety.

## Materials and methods

### Ethical approval

All experimental protocols and procedures were reviewed and approved by the Mansoura University Animal Care and Use Committee (MU-ACUC), Faculty of veterinary medicine, Egypt (Code Number : VM.PhD.23.03.10). We confirm that all methods were performed in accordance with the relevant guidelines and regulations, including the ARRIVE guidelines.

### Chemicals and reagents

Low molecular weight Chitosan (50–190 kDa, deacetylation degree 75–85%) was sourced from Sigma-Aldrich **(**St. Louis, MO, USA, Cat. No. 448869). Diagnostic kits for oxidative stress biomarkers, including malondialdehyde (MDA, Cat. No. MD 25 29), reduced glutathione (GSH, Cat. No. GR 25 11), superoxide dismutase (SOD, Cat. No. SD 25 21), and catalase (CAT, Cat. No. CA 25 17), were obtained from Biodiagnostic Co. (Dokki, Cairo, Egypt).All other reagents used in this study were of analytical grade.

### Synthesis and characterization of Nano-Chitosan (nCS)

Nano-chitosan (nCS) was prepared by the ionic gelation method according to Tang, et al^16^.. Briefly, CS solution was prepared by dissolving 3 g of CS powder in 442 mL of distilled water containing 1.5% glacial acetic acid (Cat. No. 695092, Sigma-Aldrich, St. Louis, MO, USA). The mixture was magnetically stirred for 30 min until it became opalescent. Subsequently, 50 ml of sodium tripolyphosphate (TPP, Cat. No. 238503, Sigma-Aldrich, St. Louis, MO, USA) solution was added dropwise to the CS solution under continuous stirring. The reaction was maintained for 45 min at room temperature to facilitate ionic cross-linking. The resulting nCS suspension was sonicated for 10 min and stored at 4 °C for subsequent use.

For morphological assessment, a droplet of the nanoparticle suspension was deposited onto a carbon-coated copper grid and allowed to dry at room temperature. The shape and average size of the nCS were examined using a transmission electron microscope (TEM) (JEOL JEM-2100, Japan) at an acceleration voltage of 200 kV. Particle diameters were analyzed using ImageJ software (NIH, v. 1.50i). Additionally, Dynamic Light Scattering (DLS) and zeta potential analyses were performed using a Zetasizer instrument (Malvern Instruments, UK) at a scattering angle of 90° and 25 °C. Following the procedure of Nomier, et al^17^., the nCS suspension was diluted 100-fold with double-distilled water to evaluate the hydrodynamic diameter, size distribution, and Zeta Potential of the synthesized nanoparticles.

### Fish maintenance and acclimatization

Eighty clinically healthy Nile tilapia (*o. niloticus*) (mean weight **65 ± 5 g)** were sourced from El-Manzalah Fish farm (Dakahlia, Egypt). The fish were transported to the laboratory in aerated polyethylene bags. Upon arrival, fish were screened for healthy status to ensure they were asymptomatic and free from external lesions and acclimatized for 14 days in glass tanks containing 50 L of dechlorinated tap water. Tanks were supplied with continuous aeration (BOYU S 2000 Air pump, Malaysia). During this period, Fish were fed a commercial pellet diet ad libitum twice daily with 50% daily water renewal. Feeding was ceased 24 h pre-exposure and during the 96-hour acute toxicity assay to minimize metabolic waste and nanoparticles adsorption.

### Experimental design and toxicity exposure protocol

**Fig. 1:**
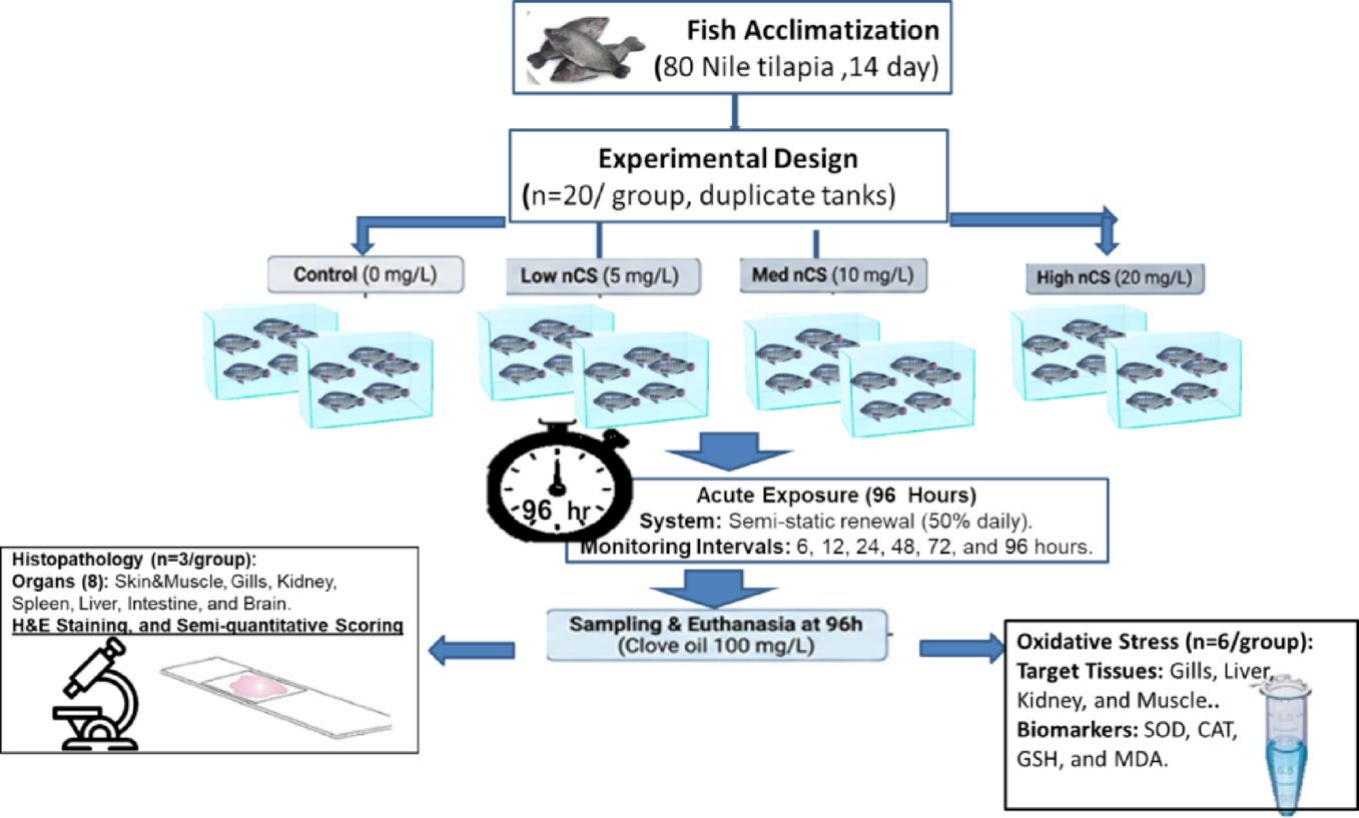
Experimental design.

The 96-hour acute toxicity assessment followed a semi-static renewal design (Fig. 1) in accordance with OECD guideline 203^18^. Following the accommodation time, fish were divided into four groups (*n* = 20/group; duplicate tanks of 10) exposed to the following nominal nCS concentrations of 0 (control), 5, 10, and 20 mg/L, based on previous studies^19^. To maintain nominal concentration and water quality, 50% of the water was renewed daily with freshly prepared nCS suspensions. Physicochemical parameters were monitored every 12 h using a multiparameter meter (HORIBA U-52, Japan) and maintained within the following ranges: pH 7.2 ± 0.1, dissolved oxygen (DO) > 5.8 mg/L, ammonia (NH3) < 0.02 mg/L, temperature 26 ± 1 °C, under a 12:12 h light: dark cycle.

Mortality and behavioral alterations were monitored and recorded at 6, 12, 24, 48, 72, and 96 h. Dead fish were immediately removed along with excreta to prevent water quality deterioration. A fish was considered dead when it showed no opercular movement or response to tactile stimuli.

### Sample collection

At the termination of the 96-hour bioassay, all remaining fish were humanely euthanized by immersion in an intentional anesthetic overdose of clove oil (100 mg/L) for 10 min^20,21^. Immediately post-mortem, fish were dissected on an ice-cold surface. Tissue samples were rapidly harvested, rinsed with cold physiological saline, and divided for histopathology and oxidative stress analysis.

### Oxidative stress markers analysis

Tissue samples (gill, liver, kidney, and muscle) from six fish per group (n = 6) were homogenized (1:10 w/v) in ice-cold phosphate-buffered saline (0.1 M PBS, pH 7.4) using a homogenizer (Omni TH-01,USA). All biochemical readings were performed in duplicate. The homogenates were centrifuged at 3500×g for 15 min at 4 °C. The resulting supernatants were collected and stored at −20 °**C** for subsequent biochemical analysis^22^. Lipid peroxidation (LPO) was estimated by measuring malondialdehyde (MDA) level colorimetrically at 534 nm. The assay is based on the reaction between MDA and thiobarbituric acid (TBA) to yield a pink-colored complex^23^. Regarding antioxidant defense, superoxide dismutase (SOD) activity was determined based on its ability to inhibit the phenazine methosulfate-mediated reduction of nitroblue tetrazolium (NBT) at 560 nm^24^. Catalase (CAT) activity was assessed colorimetrically by monitoring the rate of hydrogen peroxide (H_2_O_2_) decomposition at 510 nm, where the reaction is terminated by a catalase inhibitor and the residual H_2_O_2_ react with a chromogen^25^.additionally, reduced glutathione (GSH) content was estimated using Ellman’s reagent at 412 nm, which relies on the reduction of 5, 5’-dithiobis (2-nitrobenzoic acid) (DTNB) to a yellow compound^26^.

### Histopathological assessment

Tissue specimens from eight different organs including skin, muscle, gills, kidney, spleen, liver, intestine, and brain (cerebellum and optic tectum) (*n* = 3 per treatment) were excised and stabilized in neutral formalin (10%) for 24 h. Standard histological processing was applied, including dehydration in alcohol, clearing in xylene, and embedding in paraffin block. Sectioning was performed at 5 μm thickness using a microtome (Leica RM 2155, England), followed by routine staining with Hematoxylin and Eosin (H&E). The stained sections were examined using a light microscope (Olympus CX 31, Shinjuku-ku, Tokyo, Japan)^27^.

### Semi-quantitative scoring and indices

Histopathological alterations were evaluated using a modified protocol based on^28^. Lesions were categorized into four reaction patterns (circulatory, regressive, progressive, and inflammatory) and graded on an ordinal scale of 0 to 3 representing the extent of tissue damage : 0 = None; 1 = Mild (focal/minimal; <25% of field); 2 = Moderate (multifocal/regional; 25–50%; clear architectural impact); 3 = Severe (diffuse/coalescing; >50%; marked architectural loss/hemorrhage/necrosis). Nine randomly selected microscopic fields per fish were examined at 100x magnification (3 sections/fish × 3 fields/section). To quantify the histopathological damage, the Organ Severity Index $\:\left({I}_{org}\right)$ was calculated for each replicate (*n* = 3) by summing the specific lesion scores$\:({I}_{org}=\sum\:lesion\:scores$). Subsequently, the data were normalized as a Percent Severity Index $\:\left({SI}_{\%}\right)$ using the formula:

$$\:{SI}_{\%}=\frac{{I}_{org}}{maximum\:possible\:score}\times\:100$$

Where the maximum possible score equals the total number of lesion types evaluated multiplied by the maximum severity score of 3.

### Statistical analysis

Data were expressed as the mean ± standard deviation (SD) (*n* = 6 fish/group for oxidative stress, and *n* = 3 fish/group for histopathology). Prior to analysis, the data were checked for normality using the Shapiro-Wilk test. The data for oxidative stress markers and histopathological indices$\:\left(\:{SI}_{\%\:}\right)\:$were analyzed using a one-way analysis of variance (ANOVA) followed by Tukey’s post-hoc test to determine significant differences between groups. Pearson correlation coefficients *(r)* were assessed to evaluate the dose-dependent relationships between nCS concentrations and oxidative stress biomarkers. A probability value of *P* < 0.05 was considered statistically significant. All analyses were performed using GraphPad Prism software (version 8.0) (GraphPad Software, Inc., USA).

## Result

### Characterization of nCS

**Fig. 2:**
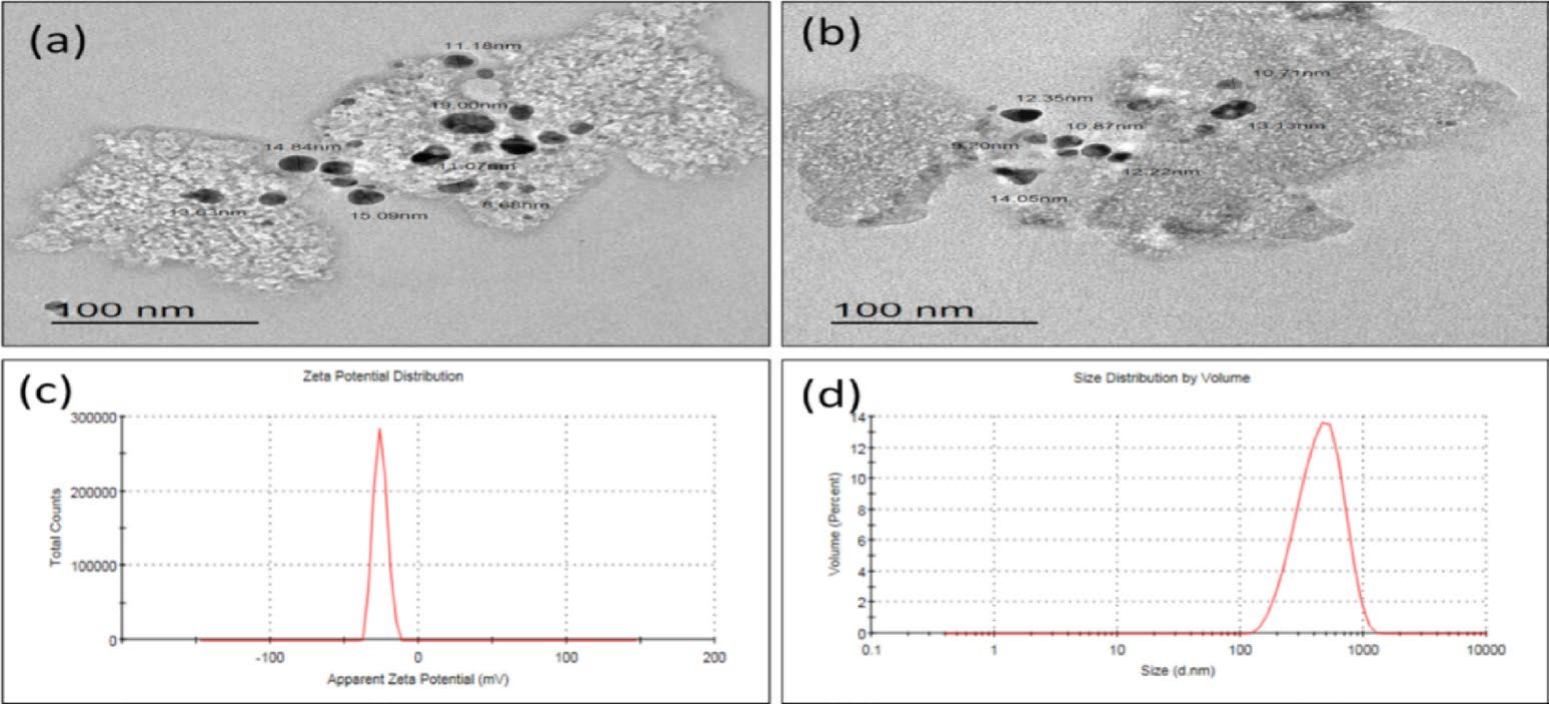
Characterization of nCS. **(a, b)** TEM images showing the morphology and primary particle size of nCS (scale bar = 100 nm). **(c)** Zeta potential distribution curve. **(d)** DLS size distribution profile by volume.

The structural and surface characteristics of the synthesized nCS were determined using TEM and DLS analyses. TEM micrographs showed roughly small, spherical, variable-sized particles and with an average size ranging from 8 to 20 nm. In an aqueous suspension, DLS analysis measured a mean hydrodynamic diameter (Z-average) of 346.9 nm. The recorded Polydispersity Index (PdI) was 0.174. Additionally, the zeta potential was measured at −25.9 mV (Fig. 2).

### Acute lethality and time-mortality response

**Fig. 3:**
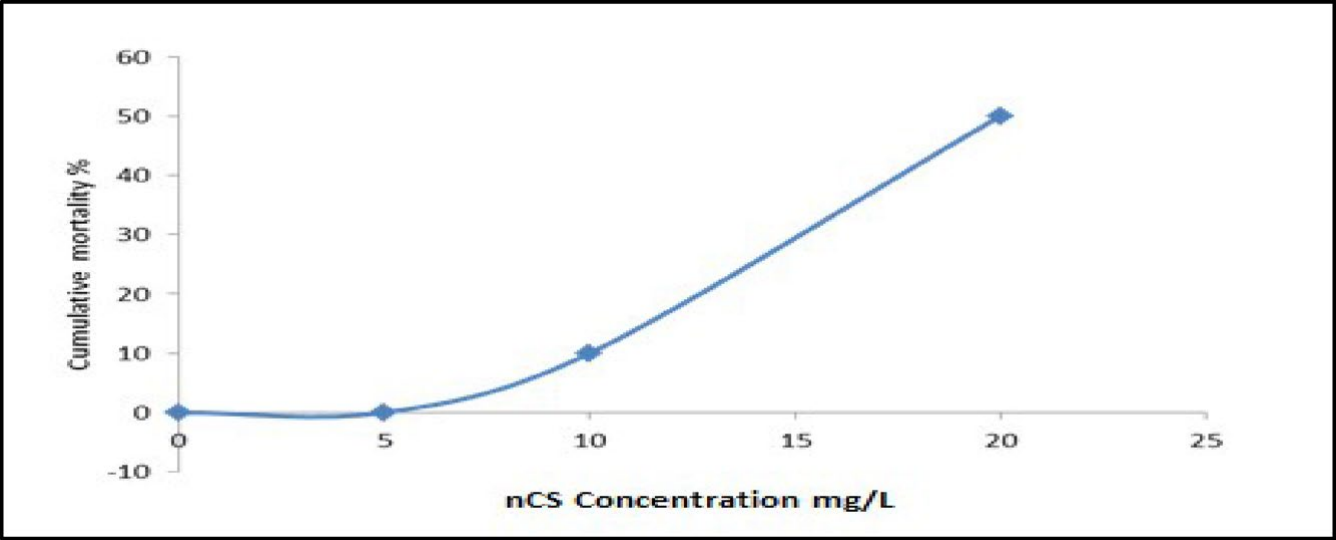
Cumulative mortality of Nile tilapia exposed to different concentration of nCS.

The lethality of waterborne nCS in Nile tilapia exhibited a clear dose and time dependent mortality pattern during the 96-hour exposure period. As illustrated in (Fig. 3), there was no mortality in the control (0 mg/L nCS) and the lowest exposure concentration (5 mg/L nCS) during the experimental duration. At the intermediate concentration (10 mg/L nCS), mortality onset occurred at 72 h, with a cumulative mortality of 10% (2/20fish). In contrast, the highest concentration (20 mg/L nCS) induced earlier and more severe mortality, with death first observed at 48 h (10%, 2/20). The mortality rate climbed to 30% (6/20) by 72 h and reached 50% (10/20) at the 96-hour endpoint. At concentration ≥ 10 mg/L, mortality was associated with behavioral changes, including erratic swimming, loss of equilibrium, and reduced responsiveness to external stimuli, indicating severe neurotoxicity and systemic physiological failure.

### Assessment of oxidative status

The oxidative stress analysis revealed that nCS exposure significantly disrupted the antioxidant defenses of Nile tilapia in a dose-dependent manner. All examined tissues (gills, kidney, liver, and muscle) showed enzymatic depletion and lipid peroxidation, reflecting a profound state of cellular stress with severity increasing alongside nCS concentration.

### Lipid oxidation

**Fig. 4:**
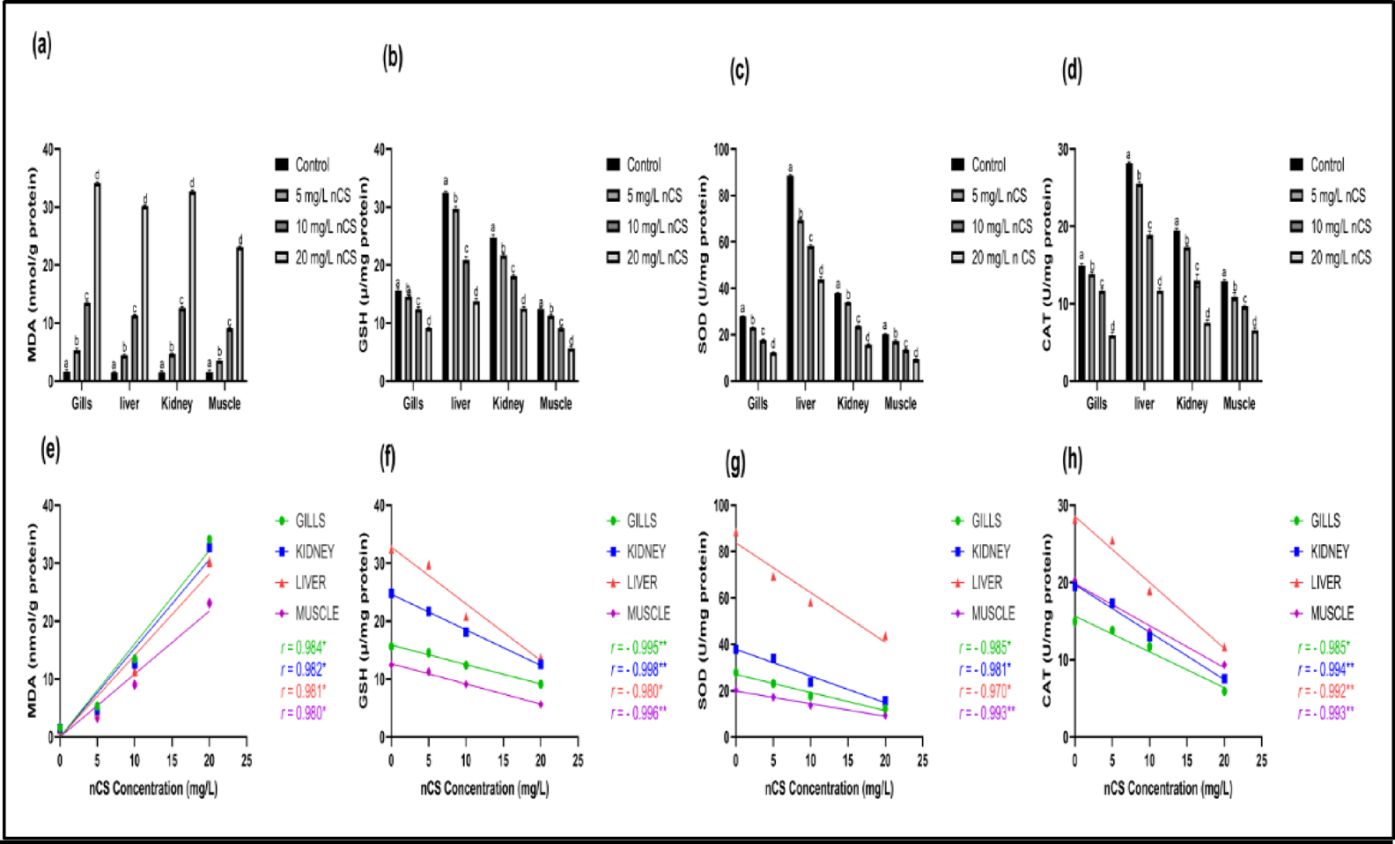
Oxidative stress biomarkers in Nile tilapia tissues following nCS exposure and their Pearson correlation analysis. **(a-d)** oxidative stress biomarkers in gills, liver, kidney, and muscle after 96 h exposure to nCS at 0 (control), 5, 10, 20 mg/L : **(a)** MDA content, **(b)** GSH content, **(c)** SOD activity, **(d)** CAT activity. Data are reported as mean ± SD (*n* = 6). Different letters indicate significant differences between doses within the same organ (*P* < 0.05). **(e-h)** Linear regression and Pearson correlation analysis between increasing nCS concentration and the respective biomarkers: **(e)** MDA, **(f)** GSH, **(g)** SOD, and **(h)** CAT. Values represent the Pearson correlation coefficient (*r*), with asterisks indicating significance: (*) for *P* < 0.05 and (**) for *P* < 0.01.

The amount of MDA in Nile tilapia tissues was substantially elevated in a dose-dependent manner after 96 h of exposure to nCS **(**Fig. 4a**).** At the low and moderate doses (5 and 10 mg/L nCS), there was a highly significant increase in MDA content (*P* < 0.0001) in all four examined tissues compared to the control group. Exposure to the maximum dose (20 mg/L nCS) resulted in a significant surge in lipid peroxidation, with the highest MDA accumulation in the gills and kidney, reflecting severe cellular membrane damage in these primary target organs.

### Antioxidant defense system responses

Over 96 h, nCS exposure led to a marked, dose-dependent reduction in enzymatic (SOD, CAT) and non-enzymatic (GSH) antioxidant levels within all four examined tissues (*P* < 0.0001) (Fig. 4b-d**).** Reduced glutathione content, alongside superoxide dismutase and catalase activity exhibited a progressive and consistent depletion across the gills, kidney, liver, and muscle as nCS dose increased from 5 to 20 mg/L. This consistent decrease indicates the exhaustion of the antioxidant defense system, making the organs highly susceptible to oxidative damage.

Correlation analysis showed a significant positive correlation between nCS concentrations and MDA levels across all examined tissues (*r* = 0.980 to 0.984(, reflecting a dose-dependent increase in lipid peroxidation (Fig. 4e). Conversely, significant negative correlations were recorded with antioxidant biomarkers, including GSH (*r* = −0.980 to −0.998), SOD (*r* = −0.970 to −0.993), and CAT (*r* = −0.985 to −0.994), showing a progressive decline in enzymatic and non-enzymatic defenses (*P* < 0.05) (Fig. 4f-h**)**.

Overall, oxidative stress changes varied by tissue: Gills ≈ kidney > liver > muscle. This pattern aligns with statistical significance, where muscle showed the least change at low concentrations (5 mg/L), and gills consistently exhibited the earliest and most severe changes across all biomarkers. Lipid peroxidation and antioxidant levels were strongly inversely correlated across all tissues.

### Histopathological alteration assessment

Histopathological analysis revealed a dose–dependent relationship between nCS concentration and the severity of pathological findings across all examined tissues in Nile tilapia. While control groups (Score 0) maintained normal histological structure, exposed groups showed increasing damage from subtle cellular changes at 5 mg/L to severe architectural damage at 20 mg/L. The semi-quantitative analysis of the percentage of maximum possible score (% Max) is summarized in Table 1, with gills, kidney, and liver showing the most damage, correlating with their roles as primary sites for xenobiotic exposure, metabolism, and excretion.

**Table 1:** Percent of maximum across nCS exposure doses.

Organ	Control	5 mg/L	10 mg/L	20 mg/L
Gills	**0.0% (± 0.0)** ^**d**^	**27.8% (± 5.6)** ^**c**^	**59.3% (± 8.5)** ^**b**^	**87.0% (± 6.4)** ^**a**^
Kidney	**0.0% (± 0.0)** ^**c**^	**25.0% (± 14.4)** ^**c**^	**55.6% (± 9.6)** ^**b**^	**86.1% (± 9.6)** ^**a**^
Liver	**0.0% (± 0.0)** ^**c**^	**24.4% (± 15.4)** ^**c**^	**51.1% (± 3.8)** ^**b**^	**84.4% (± 10.2)** ^**a**^
Intestine	**0.0% (± 0.0)** ^**c**^	**22.2% (± 9.6)** ^**c**^	**50.0% (± 14.7)** ^**b**^	**79.6% (± 8.5)** ^**a**^
Spleen	**0.0% (± 0.0)** ^**c**^	**20.0% (± 11.5)** ^**c**^	**48.9% (± 15.4)** ^**b**^	**77.8% (± 3.8)** ^**a**^
Skin & Muscle	**0.0% (± 0.0)** ^**c**^	**16.7% (± 14.4)** ^**bc**^	**44.4% (± 17.3)** ^**ab**^	**75.0% (± 16.7)** ^**a**^
Optic Tectum	**0.0% (± 0.0)** ^**c**^	**15.6% (± 3.8)** ^**c**^	**42.2% (± 10.2)** ^**b**^	**73.3% (± 11.5)** ^**a**^
Cerebellum	**0.0% (± 0.0)** ^**c**^	**13.9% (± 9.6)** ^**c**^	**38.9% (± 4.8)** ^**b**^	**72.2% (± 4.8)** ^**a**^

Data are expressed as mean ± SD (% of Max). Different superscript letters within the same row indicate.

significant differences between groups *(P < 0.05).*

### Respiratory system (Gills)

**Fig. 5:**
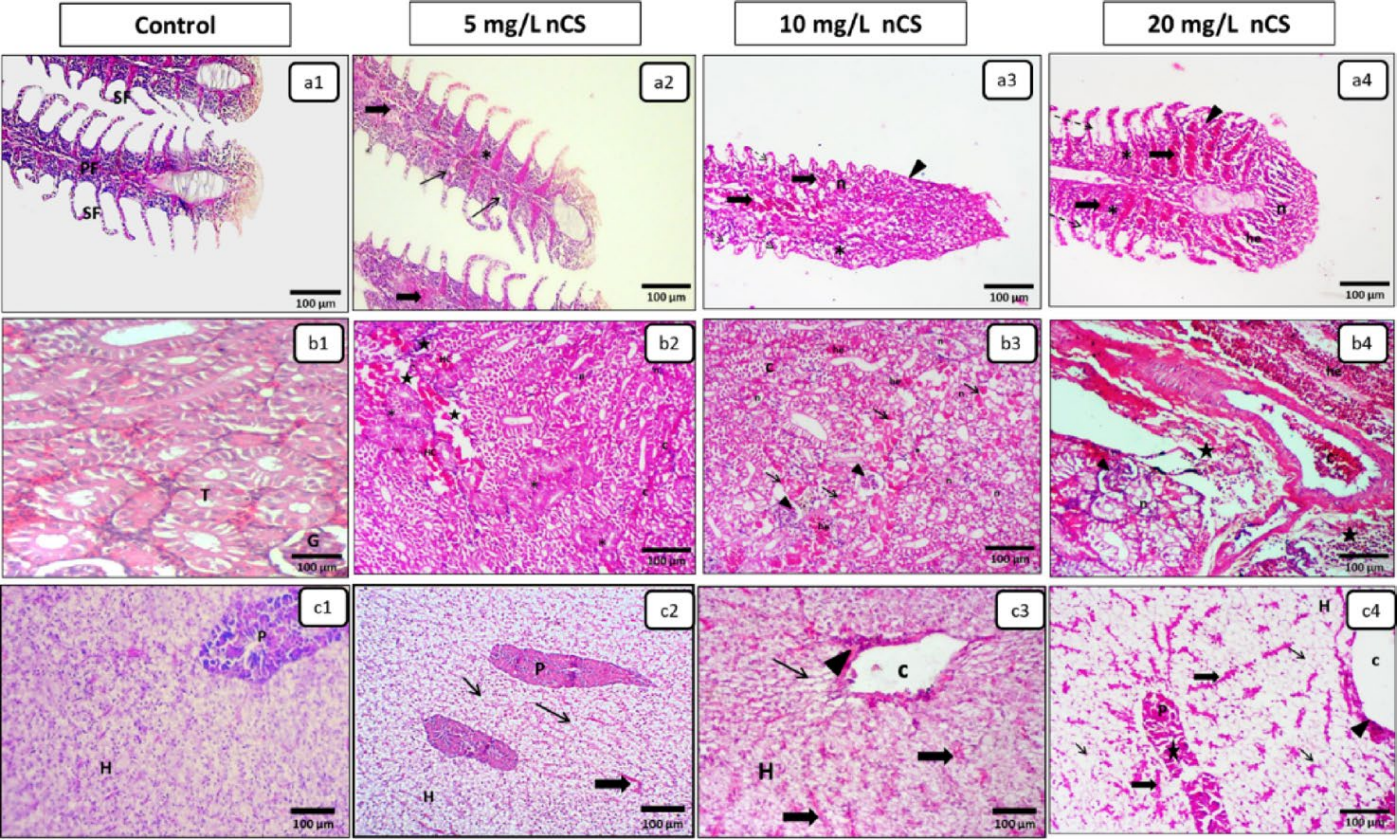
Representative photomicrographs in the gills, kidney, and liver after 96 h of nCS exposure (H&E). **(a1-a4) Gills**: Control **(a1)** showing normal primary (PF) and secondary (SF) filaments. Exposed groups **(a2-a4)** showing a dose-dependent progression: epithelial hyperplasia (asterisks) and congestion (thick arrows). Lesions progress from vacuolization (thin arrows) at **a2** to lamellar fusion (arrowhead), epithelial lifting/subepithelial edema (dashed arrows), and lamellar necrosis (n) at **a3** and **a4**, accompanied by hemorrhage (he) at **a4**. **(b1-b4) Kidney**: Control **(b1)** showing normal tubules (T) and glomerulus (G). Exposed groups **(b2-b4)** showing a dose-dependent progression: tubular necrosis (n), vascular congestion (c), and edema with inflammatory cell infiltration (stars). Lesions progress from tubular cloudy swelling (asterisk) and hyaline casts (HC) at **b2**, to hyaline droplet degeneration (thin arrows), hemorrhage (he), and glomerular tuft collapse (arrowheads) at **b3**, severe acute necrosis and shrunken glomeruli (arrowheads) at **b4**. **c1-c4) Liver**: Control **(c1)** showing normal polygonal hepatocytes (H) and exocrine pancreas (P). Exposed groups **(c2-c4)** showing a dose-dependent progression: vacuolar degeneration (thin arrows) and sinusoidal congestion (thick arrows). Damage progression with central venous congestion (c), pericentral hemorrhage (arrowheads) at **c3 and c4**, and acinar granule depletion (star) at **c4**.

The gills showed the most severe damage among all organs. The control group maintained normal gill filament architecture (Fig. 5a^1^). Conversely, the 5 mg/L group showed localized vacuolization, mild congestion, and epithelial hyperplasia of primary filaments (Fig. 5a^2^). In the 10 mg/L group, the lesions progressed to focal lamellar fusion and scattered epithelial lifting accompanied by focal lamellar necrosis (Fig. 5a^3^). At the highest concentration (20 mg/L), severe diffuse branchitis was observed, characterized by complete obliteration of interlamellar spaces and widespread necrotic zones (Fig. 5a^4^), which critically impairs respiratory gas exchange and osmoregulatory functions.

### Excretory system (Kidney)

The renal tissue of the control group presented a normal histological appearance with intact glomeruli and tubules (Fig. 5b^1^). Exposure to 5 mg/L induced mild, multifocal cloudy swelling of the tubular epithelium and scattered hyaline casts, alongside patchy peritubular edema and minimal inflammatory cell infiltration (Fig. 5b^2^). At 10 mg/L, the severity of the lesions increased to moderate eosinophilic tubular hyaline droplet degeneration, tubular necrosis, and prominent glomerular tuft collapse (Fig. 5b^3^). The pathological impact peaked at 20 mg/L, where severe acute necrosis, interstitial edema with dense inflammatory aggregates, and extensive focal congestion and hemorrhage (Fig. 5b^4^), indicating acute renal failure.

### Hepato-pancreatic system (Liver & Pancreas)

The hepatopancreatic tissues of the control group maintained normal polygonal hepatocytes and pancreatic acini **(**Fig. 5c^1^**)**. Exposure groups showed changes ranging from mild cytoplasmic vacuolization and vascular congestion (5 mg/L) **(**Fig. 5c^2^**)** to marked pericentral vacuolation and vascular congestion, accompanied by focal pericentral hemorrhage (10 mg/L) **(**Fig. 5c^3^**).** At the 20 mg/L, diffuse and extensive hepatic vacuolization and marked sinusoidal congestion occurred, with focal pancreatic acinar granule depletion **(**Fig. 5c^4^**)**, suggesting severe metabolic dysfunction and lipid accumulation.

### Digestive system (intestine)

**Fig. 6:**
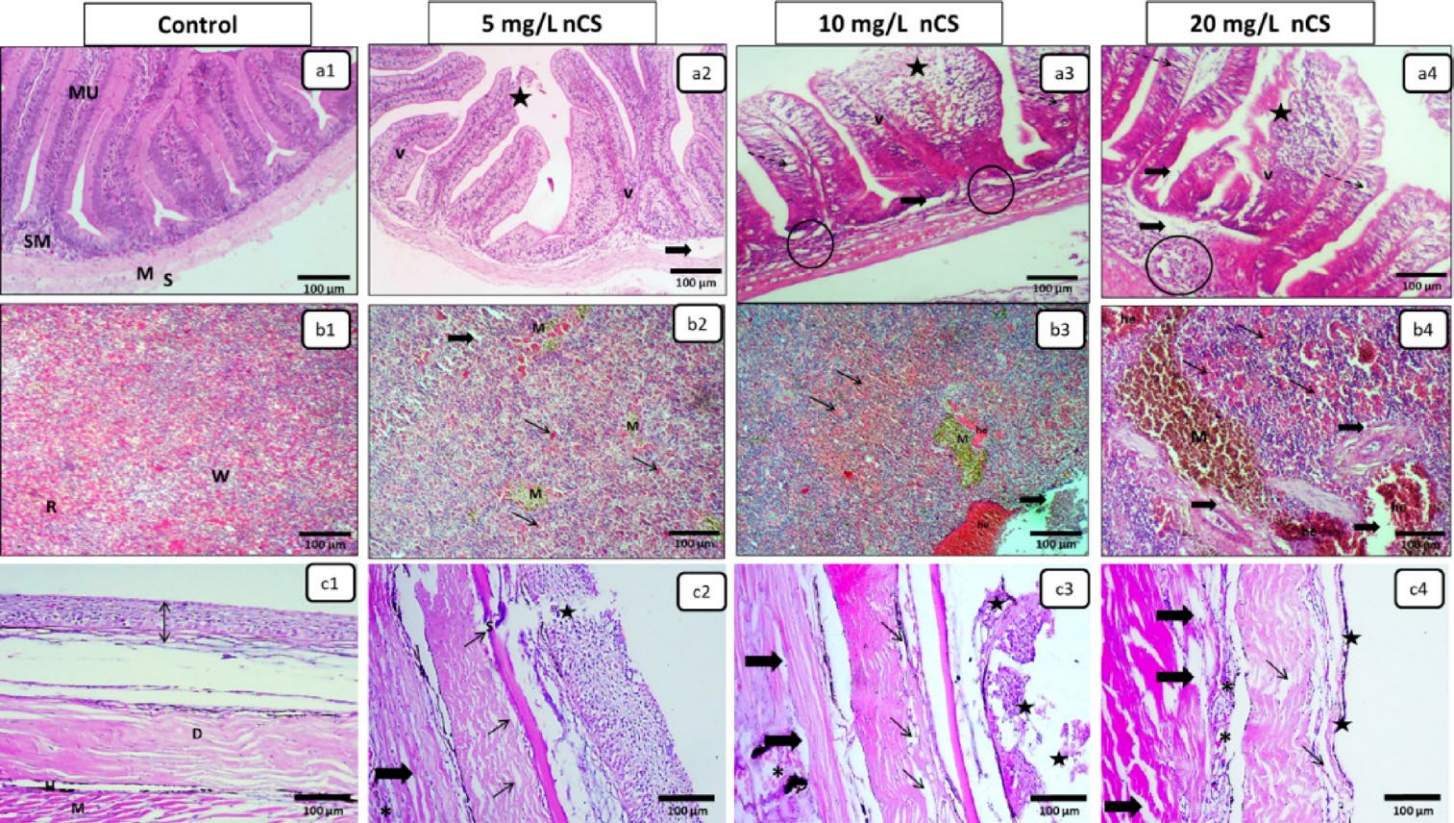
Representative photomicrographs in the intestine, spleen, and skin with underlying dorsal musculature after 96 h of nCS exposure (H&E). **(a1-a4) Intestine**: Control **(a1)** showing normal mucosa (MU), submucosa (SM), tunica muscularis (M), and tunica serosa (S). Exposed groups **(a2-a4)** showing a dose-dependent progression: shortened/fused villi (V) and epithelial sloughing/necrosis (stars), and submucosal/lamina propria edema (thick arrows), accompanied by congested vessels with inflammatory cells (circles) and enterocytic vacuolation (dashed arrows) at **a3 and a4. (b1-b4) Spleen**: Control **(b1)** showing normal red pulp (R) and white pulp (W). Exposed groups **(b2-b4)** showing a dose-dependent progression: red pulp congestion (thin arrows), white pulp lymphoid depletion) thick arrows), and prominent melano-macrophage centers (M), accompanied by hemorrhage (he) at **a3 and a4. (c1-c4) Skin and musculature**: Control **(c1)** showing normal epidermis (double arrow), dermis (D), hypodermis (H), and musculature (M). Exposed groups **(c2-c4)** showing a dose-dependent progression: epidermal and scale (S) necrosis/erosion (stars) and dermal edema (thin arrows), leukocytic infiltration (asterisks), myofiber degeneration with inter-fiber edema (thick arrows).

The intestinal architecture of the control group preserved normal intestinal layers (Fig. 6a^1^). At 5 mg/L, mild villus blunting and focal epithelium sloughing were the primary observations (Fig. 6a^2^). As the dose increased to 10 mg/L, the intestinal lesions included villous apical necrosis and enterocytic vacuolation along with marked submucosal congestion and leukocytic infiltration (Fig. 6a^3^). The 20 mg/L group manifested severe necrotic enteritis, characterized by extensive epithelial sloughing that exposed the edematous lamina propria (Fig. 6a^4^), severely compromising nutrient absorption and mucosal barrier integrity.

### Immune system (Spleen)

The splenic parenchyma of the control group showed a normal appearance of red and white pulp (Fig. 6b^1^). The 5 mg/L exposure resulted in mild red pulp congestion and scattered pinpoint hemorrhage (Fig. 6b^2^). The 10 mg/L dose progressed to moderate red pulp congestion and white pulp depletion with multifocal hemorrhage (Fig. 6b^3^). The severe disruption of splenic architecture occurred in the 20 mg/L dose and manifested by extensive red pulp congestion and marked white pulp depletion, alongside diffuse melano-macrophage centers prominence (Fig. 6b^4^), suggesting marked immunosuppression and hematopoietic disruption.

### Integumentary and dorsal musculature system

Control specimens showed intact skin layers and attachment musculature (Fig. 6c^1^). At 5 mg/L, focal epidermal sloughing occurred, but myofibers mostly remained intact (Fig. 6c^2^). However, as exposure increased to 10 mg/L, these lesions progressed to multifocal epidermal erosion accompanied by moderate interstitial dermal and initial muscle fibers degeneration (Fig. 6c^3^). These pathological continuums culminated at 20 mg/L with diffuse epidermal necrosis and complete sloughing, with underlying dorsal musculature showing acute degenerative changes, including myofiber fragmentation and significant interfibrillar edema (Fig. 6c^4^), compromising the primary physical defense barrier.

### Nervous system (Optic tectum and Cerebellum)

**Fig. 7:**
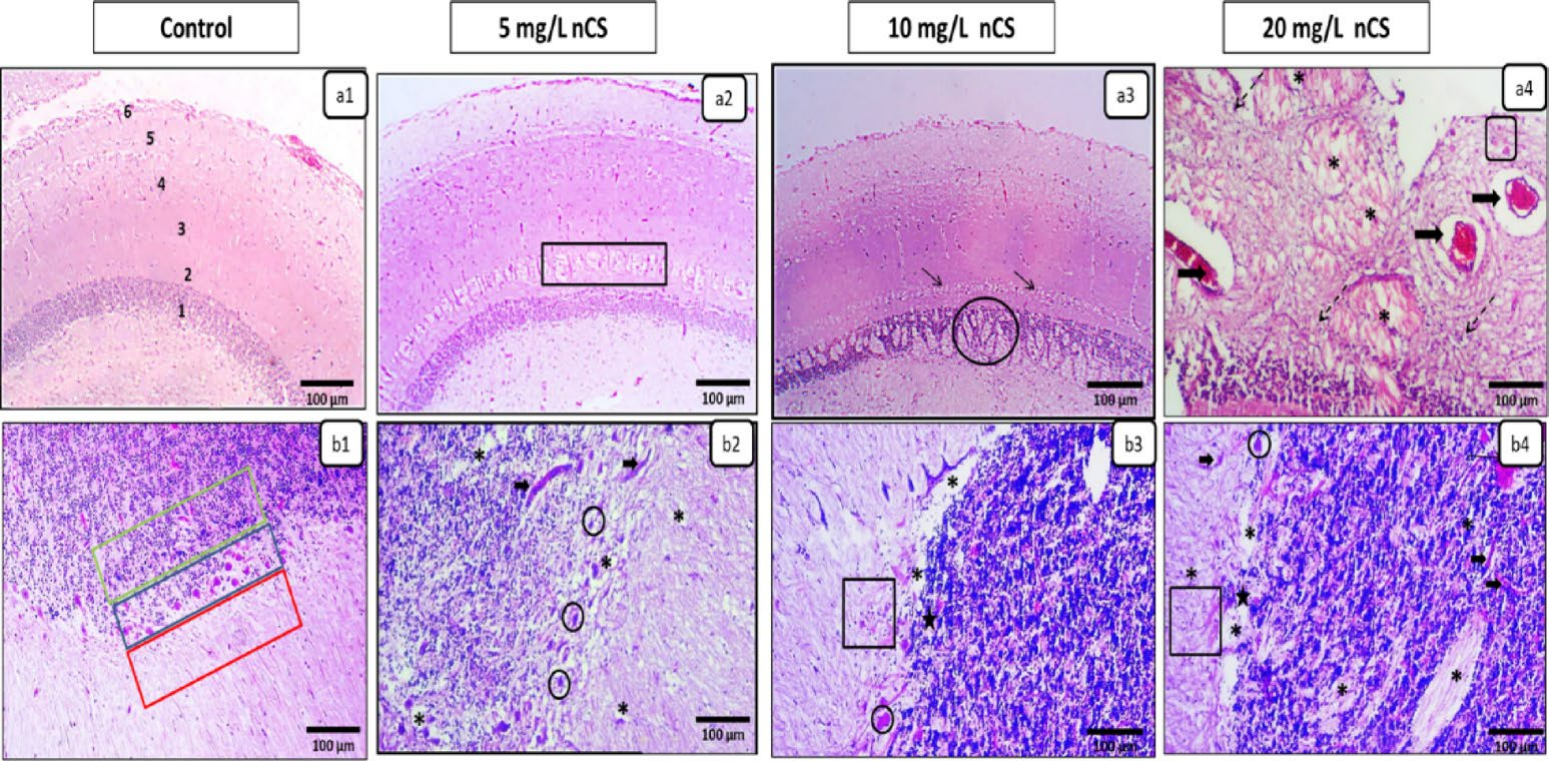
Representative photomicrographs in the brain (optic tectum and cerebellum) after 96-hour nCS exposure (H&E). **(a1-a4) Optic Tectum**: Control **(a1)** showing six normal layers: (1) Stratum periventriculare (SPV), (2) Str. album centrale (SAC), (3) Str. griseum centrale (SGC), (4) Str. fibrosum et grisium superficiale (SFGS), (5) Str. opticum (SO), and (6) Str. marginale (SM). Exposed groups **(a2-a4)** showing a dose-dependent progression of vacuolation: starting mild in the SAC (rectangle) at **a2**, becoming widespread in the SPV (circle) with necrosis in granular cells (thin arrows) at **a3**, and progressing to extensive spongiform vacuolation with shrunken neurons across all layers (asterisks), multifocal vascular congestion and perivascular edema (thick arrows), eosinophilic granular cell aggregation (square), and diffuse gliosis (dashed arrows) at **a4**. **(b1-b4) Cerebellum**: Control **(b1)** showing a normal three-layered cortex: molecular (red box), Purkinje (blue box), and granular (green box) layers. Exposed groups **(b2-b4)** showing a dose-dependent progression of neuropil rarefication/spongiosis (asterisks) and neuronal shrinkage/necrosis (circles), vascular congestion and perivascular edema (thick arrows) at **b2 and b4**, granular layer dispersion (stars) and reactive gliosis (square) at **b3 and b4**, accompanied by focal hemorrhage (thin arrow) at **b4**.

Control specimens exhibited normal histoarchitecture; the optic tectum displayed six intact layers with clear cellular organization and intact neuropil (Fig. 7a^1^), while the cerebellum showed well-demarcated layers with typical cellular density and structural integrity (Fig. 7b^1^). At 5 mg/L, mild diffuse vacuolation was observed in the Stratum Album Centrale (SAC) of the optic tectum (Fig. 7a^2^), whereas the cerebellum showed minimal neuropil rarefication with shrunken neurons (Fig. 7b^2^). Fish exposed to 10 mg/L exhibited more pronounced pathology, including widespread neuropil vacuolation and spongy degeneration predominantly affecting the Stratum periventriculare (SPV) of the optic tectum (Fig. 7a^3^) and the cerebellar granular layer (Fig. 7b^3^). The highest dose (20 mg/L) induced severe neurotoxic lesions characterized by extensive spongiform changes throughout tectal layers with marked neuronal necrosis (Fig. 7a^4^). Cerebellar sections revealed granular cell dispersion, prominent reactive gliosis, and perivascular edema (Fig. 7b^4^).

**Fig. 8:**
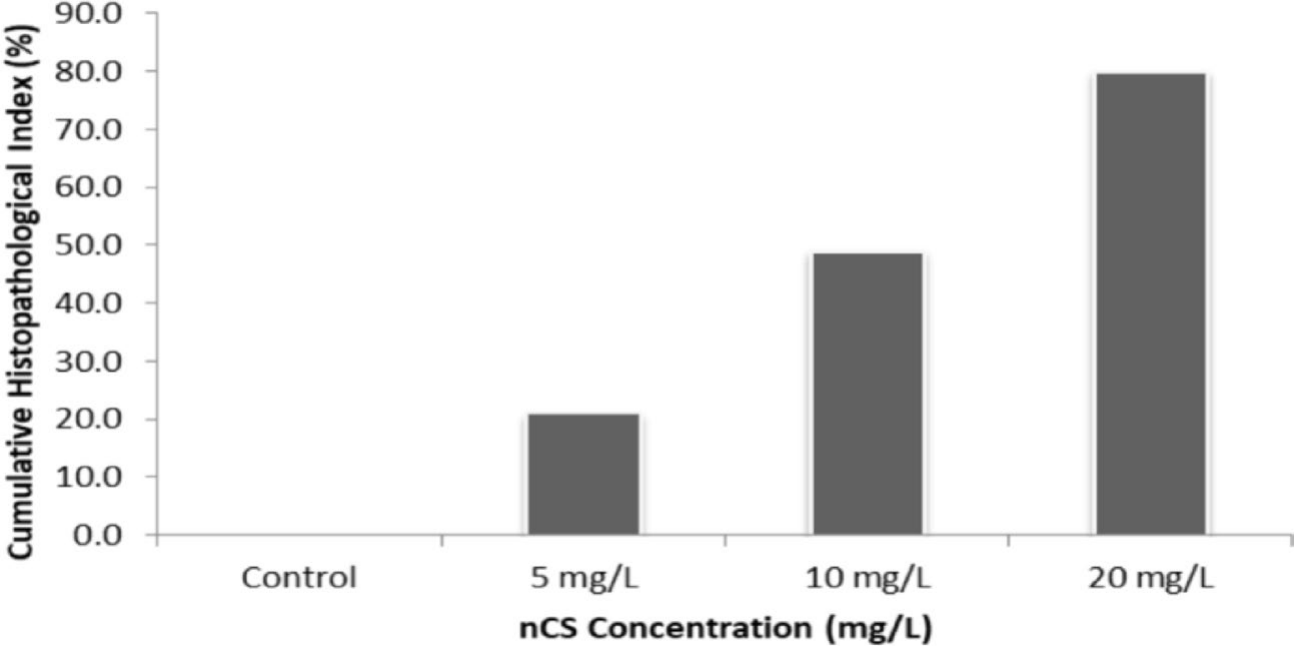
Dose-dependent increase in the cumulative histopathological index of *Orechromis niloticus* following nCS exposure.

The cumulative histopathological index, reflecting the mean damage percentage across all eight organs, increased significantly from 0.0% in the control group to 21%, 49%, and 80% in the 5, 10, and 20 mg/L groups, respectively **(**Fig. 8**)**, confirming a dose-dependent systemic toxicity.

## Discussion

The present study provides a comprehensive assessment of the acute toxicity of nCS in Nile tilapia, addressing the need to understand its safety relative to its potential benefits. A recent study has demonstrated the dietary application of nCS for enhancing growth and immunity, particularly in Nile tilapia^29^. However, our results demonstrate that waterborne exposure presents a distinct safety challenge, where therapeutic potential is strictly dose-dependent. Exposure induced a critical transition from biocompatibility at lower concentration to severe systemic toxicity and mortality at higher concentrations, indicating a limited safety margin for nCS application. Aquaculture practices commonly use 5 mg/L as an initial dose for direct water treatments such as ammonia removal^30^. Consistent with this, our research indicates that LMW nCS should not exceed a maximum safe threshold of < 5 mg/L, as concentrations above this level may cause systemic toxicity due to the nanoparticles’ ultra-small size and high bioavailability. This acute toxicity is attributed to a collapse in antioxidant defenses, resulting in severe oxidative damage, with neurotoxicity and branchial necrosis identified as primary causes of death.

The observed lethality results from the combined effect of the ultra-small size of the synthesized nCS and the waterborne route of exposure. Unlike dietary exposure, which binds particles within a food matrix, waterborne exposure allows for simultaneous attack on multiple biological systems. The high surface area-to-volume ratio suggests a high potential for rapid penetration across the gill epithelium and blood-brain barrier (BBB), explaining the observed neurobehavioral anomalies prior to death. Concurrent direct ingestion of nanoparticles via osmoregulatory drinking water exposes the gut to free-floating nanoparticles, causing severe necrotic enteritis, confirmed by histopathology. This multi-target toxicity differs from previous studies showing that larger chitosan nanoparticles (84.86 nm) required high doses (280 mg/L)to induce toxicity in zebrafish^31^, and that larger dietary bulk chitosan nanoparticles (250–300 nm) act as an immunostimulant^32^. Our results demonstrate that waterborne exposure to ultra-small particles bypasses these defenses, leading to increased bioavailability and toxicity resembling nanotoxic models for metals like silver (16.6 nm)^33^ and copper (80 nm)^34^.

The enhanced bioavailability and systemic toxicity are attributed to the synthesized nCS’s physicochemical properties. Characterization revealed a dual-scale behavior characteristic of polymeric nanomaterials. DLS showed a mean hydrodynamic diameter of 346.9 nm, indicating the presence of a hydration layer and stable nanocluster formation in water. TEM confirmed the presence of ultra-small primary particles (8–20 nm) within the dehydrated solid core^35^. The low PdI (0.174) verified that the suspension was highly uniform and monodisperse, eliminating the possibility of random, unstable aggregation^35^. The zeta potential (−25.9 mV) was critical in the waterborne exposure model, resulting from the ionic gelation neutralizing chitosan’s cationic nature and creating a stable negative surface charge^36^. This electrostatic repulsion maintained colloidal stability, ensuring continuous exposure of fish to non-precipitating nanoparticles^36^.

The toxicity of nCS in aquatic organisms is unexplored compared to other nanomaterials. Current studies have primarily focused on their synthesis and functionalization, neglecting ecotoxicological impacts^4^. For instance, while previous studies demonstrated that nCS induce dose-dependent developmental toxicity and mortality in zebrafish^19^, their effects on oxidative stress markers and tissue integrity were not comprehensively evaluated. Addressing this gap, our study demonstrates that oxidative stress is the primary mechanism underlying nCS-induced cytotoxicity^37^. Exposure to nCS triggered a significant disruption in the oxidant/antioxidant balance across all examined tissues. The ultra-small size (8–20 nm) of the synthesized nCS likely allows the particles to enter cells easily, such as through caveolae-mediated endocytosis, potentially overcoming electrostatic repulsion^38^. Once intracellular, nCS likely accumulates in the mitochondria, possibly leading to a reduction in the mitochondrial membrane potential. This mitochondrial stress is considered a primary cause for the massive release of reactive oxygen species (ROS)^39^. Excessive ROS generation overwhelmed cellular defenses, evidenced by depleted enzymatic (SOD, CAT) and non-enzymatic (GSH) antioxidants^40^. Because GSH plays a vital role in detoxifying ROS^41^, its depletion serves as a critical indicator of severe cellular vulnerability. Consequently, this led to severe lipid peroxidation across all tissues, confirmed by a dose-dependent elevation of MDA as a key marker of oxidative damage, indicating massive structural cellular injury^42^.

Notably, the severe depletion of antioxidant enzymes in our study closely resembles the toxicity patterns seen in teleost models exposed to high doses of silver nanoparticles (Ag-NPs)^15,43^, confirming that the high bioavailability of ultra-small nanoparticles leads to irreversible tissue damage. In contrast to dietary supplementation, which is buffered by food and stimulates antioxidant defenses^44^, waterborne exposure delivers highly bioavailable^45^, unbound nCS directly to biological interfaces. This causes severe oxidative injury that primarily targets the main uptake and systemic filtration sites.

Histopathological evaluations corresponded with the systemic antioxidant failure. These structural changes highlight the systemic toxicity mechanisms of waterborne nCS, rather than just indicating localized injury. In the gills, initial adaptive mechanism such as epithelial hyperplasia acted to increase the diffusion distance and minimize toxin uptake^46^. However, these compensatory mechanisms rapidly collapsed into diffuse branchitis and lamellar necrosis in the highest nCS concentrations. This structural damage impaired respiratory gas exchange and osmoregulatory functions^47^. Alongside branchial damage, the intestinal architecture was severely compromised due to the direct ingestion of nanoparticles via drinking. The resulting necrotic enteritis disrupts the epithelial barrier, increasing permeability and facilitating the translocation of particles into the circulatory system^48^. This aligns with findings on microplastics and Ag-NPs, which compromise the intestinal mucosal barrier and tight junctions of epithelial cells^48,49^.

Following systemic absorption, the primary organs responsible for metabolism and excretion (hepatopancreatic system and kidney) showed severe degenerative and necrotic changes, including prominent vacuolar degeneration and glomerular collapse. This destruction paralleled oxidative stress marker depletion, indicating impaired detoxification and metabolic dysfunction. The prominence of vacuolar degeneration suggests lysosomal dysfunction as a key toxicity pathway^50^, explaining the dose-dependent mortality previously reported in zebrafish models^19^.

Furthermore, the spleen representing the primary immune organ, showed significant structural damage. The marked loss of white pulp highlights a state of immunosuppression, likely due to a sharp drop in lymphocyte numbers following nanoparticle-induced toxicity^51^. Diffuse proliferation of MMCs, the main scavengers for toxins and cellular debris in fish, indicates high physiological stress and a hyperactive but exhausted immune response attempting to clear necrotic debris^52^. This severe impact on immune organs compromised the fish defenses, contributing to overall multisystem failure^53^.

Beyond internal organ damage, the progressive structural breakdown of the integumentary system compromised osmoregulation and the primary physical defense barrier^54^, leaving the fish highly susceptible to osmotic stress and secondary infection^55^. Simultaneously, the degeneration of underlying muscle fiber indicates the systemic dissemination of cytotoxicity^56^. This severely impaired swimming performance and escape ability^57^, accelerating exhaustion and mortality.

A key finding of our study is the direct evidence of nCS neurotoxicity. While nCS is commonly used as a safe drug-delivery vehicle, our results show that environmental exposure to uncoated nCS caused severe brain damage. The ultra-small nCS particles likely facilitated potential BBB penetration, inducing severe spongiform changes, reactive gliosis, and neuronal necrosis in the optic tectum and cerebellum^58,59^. This is consistent with size-dependent BBB penetration reported for other nanoparticles in fish, where particles specifically in the 20 nm range^60^ and up to 50 nm^61^ can penetrate into the fish brain. Because the optic tectum and cerebellum are the key centers for sensory integration, movement tracking, posture, and motor coordination in cichlid fish^62,63^, this localized neuro-degeneration helps explain the erratic swimming and abnormal behaviors we observed during exposure. This observation aligns with previous studies showing that nanoparticle accumulation in the teleost brain causes structural damage and behavioral disorders^64^.

While this study establishes a critical baseline, it has limitations that influence the interpretation of our findings. First, the acute 96-hour waterborne exposure captures only immediate, high-stress physiological responses, meaning our results cannot predict chronic toxicity, bioaccumulation, or long-term adaptation. Second, our strictly controlled laboratory conditions lack complex environmental factors (such as organic matter), which could alter nanoparticle agglomeration and potentially reduce their real-world bioavailability compared to our findings. Third, while our biochemical and histopathological markers provide strong evidence of systemic injury, the lack of molecular data limits our understanding of the specific genetic signaling pathways driving this toxicity. Furthermore, although oxidative stress was evident, ROS were not directly quantified, and nCS biodistribution and tissue accumulation require further characterization. Finally, without evaluating fish recovery after exposure, it remains unclear whether the observed severe tissue damage is reversible once the stressor is removed. To address these gaps, future research must incorporate chronic low-dose exposure models, include comparative controls (such as bulk chitosan) to better understand the relative size-dependent toxicity, quantify ROS generation using direct assays (e.g., DCFDA) and track nCS within target organs via advanced imaging techniques (e.g., fluorescence or electron microscopy), simulate natural aquaculture water parameters, utilize transcriptomic analyses to identify molecular mechanisms, and include post-exposure recovery periods to comprehensively assess the ecological risks of nCS.

In conclusion, this study indicates that acute 96-hour waterborne exposure to ultra-small nCS (8–20 nm) induces dose-dependent toxicity in Nile tilapia, with a 50% mortality observed at 20 mg/L. Acute exposure to concentration ≥ 10 mg/L significantly alters antioxidant defenses, leading to a marked imbalance between lipid peroxidation and antioxidant levels. This oxidative stress is closely associated with widespread histopathological damage across multiple organs. Based on these acute findings, nCS concentration < 5 mg/L showed minimal toxicity specifically for waterborne exposure under strictly controlled laboratory conditions. However, further studies on chronic exposure, bioaccumulation, and environmental dynamics are needed to determine safe thresholds for nCS application in aquaculture. The schematic diagram of the study is provided in the Supplementary Material (Supplementary Fig. S1).

## Supplementary Information

Below is the link to the electronic supplementary material.

Supplementary Material 1

## Data Availability

The data that support the findings of this study are available from the authors upon reasonable request.
