# Supplementary figures and images for: Dose-dependent acute toxicity of chitosan nanoparticles with dual assessment of multisystem toxicopathology and oxidative stress biomarkers in Nile tilapia
Source: Sci Rep. 2026 Jun 4;16:17332. doi: 10.1038/s41598-026-54988-x (PMC13236982; doi:10.1038/s41598-026-54988-x)

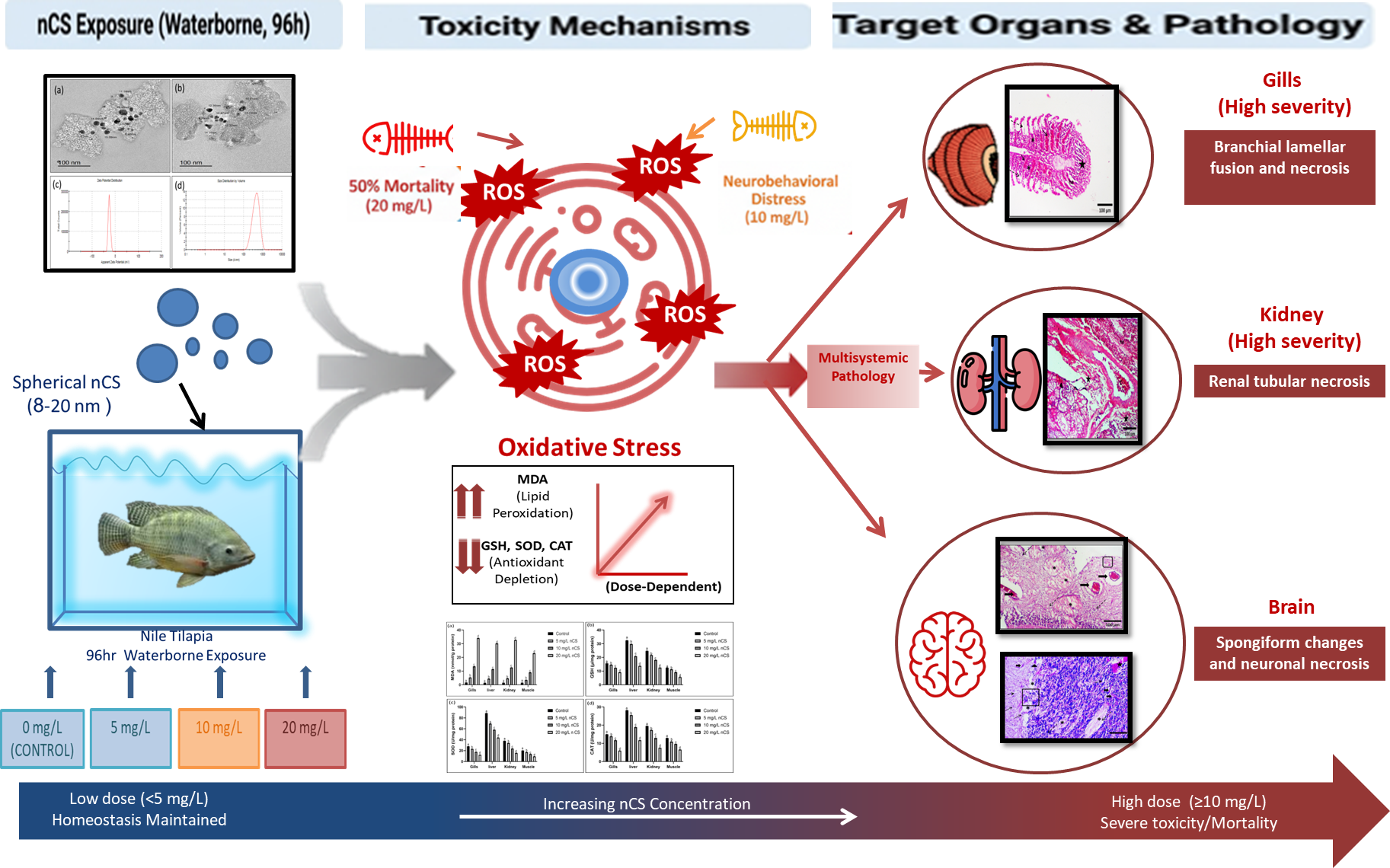

Supplement: Supplementary file 1 — Supplementary Material 1 [file 41598_2026_54988_MOESM1_ESM.png]
